# A NOVEL METABOLITE COMPOSITE SCORE EXPLAINS THE HIGHER MORTALITY ASSOCIATED WITH FRAILTY AMONG OLDER BLACK MEN

**DOI:** 10.1093/geroni/igz038.1254

**Published:** 2019-11-08

**Authors:** Megan M Marron, Tamara B Harris, Robert M Boudreau, Steven C Moore, Jason L Sanders, Stacy G Wendell, Joseph M Zmuda, Anne B Newman

**Affiliations:** 1 University of Pittsburgh, Pittsburgh, Pennsylvania, United States; 2 National Institute on Aging, Bethesda, Maryland, United States; 3 National Institutes of Health, Rockville, Maryland, United States; 4 Brigham and Women’s Hospital, Boston, Massachusetts, United States; 5 Department of Epidemiology University of Pittsburgh; Pittsburgh, Pennsylvania, United States

## Abstract

Frailty is more prevalent among black versus white older Americans. We previously sought to better characterize frailty among 287 black men ages 70-81 by identifying 37 plasma metabolites associated with vigor to frailty using the scale of aging vigor in epidemiology (SAVE). Using this information, we developed a metabolite score to determine if it explained the frailty-associated higher mortality. The Human Metabolome Database classified the metabolites as organic acids/derivatives (m=14), lipids/lipid-like molecules (m=12), organoheterocyclic compounds (m=4), benzenoids (m=3), organic nitrogen compounds (m=2), organic oxygen compounds (m=1), and nucleosides/nucleotides/analogues (m=1). Values for each were ranked into tertiles. The metabolite tertile associated with more vigorous SAVE scores was given a score of 0, the metabolite mid-tertile a score of 1, and the metabolite tertile associated with frailer SAVE scores a score of 2. The metabolite composite score was calculated as the sum of the metabolite tertile scores. One standard deviation frailer SAVE was associated with 30% higher mortality (p=0.0002), adjusting for age and study site. The association between frailty and mortality was attenuated by 56% after additionally adjusting for the metabolite score, where organic acids/derivatives and lipids/lipid-like molecules (mostly amino acids, glycerophospholipids, sphingolipids) accounted for most of the attenuation. In this model, one standard deviation higher metabolite score was associated with 46% higher mortality (p<0.0001). The metabolite score also predicted mortality among 48 community-dwelling (96% white) older men (p=0.03). These metabolites provide a deeper characterization of frailty that reproducibly explains a substantial portion of the vulnerability to death in these older men.

